# Clinical outcomes of empirical high-dose meropenem in critically ill patients with sepsis and septic shock: a randomized controlled trial

**DOI:** 10.1186/s40560-020-00442-7

**Published:** 2020-04-15

**Authors:** Tospon Lertwattanachai, Preecha Montakantikul, Viratch Tangsujaritvijit, Pitsucha Sanguanwit, Jetjamnong Sueajai, Saranya Auparakkitanon, Pitchaya Dilokpattanamongkol

**Affiliations:** 1 Department of Pharmacy, Faculty of Pharmacy, Mahidol University, Bangkok, 10400 Thailand; 2 Department of Critical Care Medicine, Faculty of Medicine, Ramathibodi Hospital, Mahidol University, Bangkok, Thailand; 3 Piyavate Hospital, Bangkok, Thailand; 4 Department of Emergency Medicine, Faculty of Medicine, Ramathibodi Hospital, Mahidol University, Bangkok, Thailand; 5 Toxicology Laboratory, Department of Pathology, Faculty of Medicine, Ramathibodi Hospital, Mahidol University, Bangkok, Thailand

**Keywords:** Meropenem, High dose, Sepsis, Septic shock, Empirical therapy, Clinical outcome

## Abstract

**Background:**

Appropriate antimicrobial dosing is challenging because of changes in pharmacokinetics (PK) parameters and an increase in multidrug-resistant (MDR) organisms in critically ill patients. This study aimed to evaluate the effects of an empirical therapy of high-dose versus standard-dose meropenem in sepsis and septic shock patients.

**Methods:**

We performed a prospective randomized open-label study to compare the changes of modified sequential organ failure assessment (mSOFA) score and other clinical outcomes of the high-dose meropenem (2-g infusion over 3 h every 8 h) versus the standard-dose meropenem (1-g infusion over 3 h every 8 h) in sepsis and septic shock patients. Patients’ characteristics, clinical and microbiological outcomes, 14 and 28-day mortality, vasopressor- and ventilator-free days, intensive care unit (ICU) and hospital-free days, percent of the time of antibiotic concentrations above the minimum inhibitory concentration (%T>MIC), and safety were assessed.

**Results:**

Seventy-eight patients were enrolled. Median delta mSOFA was comparable between two groups (– 1 in the high-dose group vs. – 1 in the standard-dose group; *P* value = 0.75). There was no difference between the two groups regarding clinical and microbiological cure, 14- and 28-day mortality, vasopressor- and ventilator-free days, and ICU- and hospital-free days. In patients admitted from the emergency department (ED) with a mSOFA score ≥ 7, the high-dose group demonstrated significantly better microbiological cure compared with the standard-dose group (75% (9/12 patients) vs. 20% (2/10 patients); *P* value = 0.03). Likewise, the high-dose group presented higher microbiological cure rate in patients admitted from ED who had either APACHE II score > 20 (83.3% (10/12) vs. 28.6% (2/7); *P* value = 0.045) or on mechanical ventilator (87.5% (7/8) vs. 23.1% (3/13); *P* value = 0.008) than the standard-dose group. Adverse events were comparable between the two groups.

**Conclusions:**

Empirical therapy with the high-dose meropenem presented comparable clinical outcomes to the standard-dose meropenem in sepsis and septic shock patients. Besides, subgroup analysis manifested superior microbiological cure rate in sepsis or septic shock patients admitted from ED.

**Trial registration:**

ClinicalTrials.gov, NCT03344627, registered on November 17, 2017

## Background

Sepsis is described as a serious organ dysfunction which results from response of dysregulated host to infection. This syndrome is an important healthcare problem that affects significant morbidity and mortality [1]. Early identification of sepsis and aggressive management in first hour are cornerstones for improving patient outcomes [2]. Delay in an initial appropriate antimicrobial therapy has been correlated with an increase in mortality [3]. The initial selection of appropriate antimicrobial is defined in terms of broad-spectrum and adequate dosing of selected antimicrobial for suspected pathogens [3]. However, appropriate antimicrobial dosing is challenging because of altered pharmacokinetics (PK) and increased multidrug-resistant (MDR) organisms in critically ill patients [4]. A larger volume of distribution, which is caused by fluid resuscitation and capillary leak syndrome, culminates in subtherapeutic levels of antimicrobial [5]. The fluctuation of renal clearance, such as augmented renal clearance or acute kidney injury, could affect by increasing or decreasing antimicrobials concentrations [6]. In addition, Suwantarat et al. reported a high prevalence of MDR gram-negative bacteria in Thailand, including 28.7% for carbapenem-resistant *Pseudomonas aeruginosa*, 45.2% for extended-spectrum beta-lactamases (ESBL) producers, and 76.3% for carbapenem-resistant *Acinetobacter baumannii* [7]. Due to the spread of antimicrobial-resistant organisms, as well as a decline in new antimicrobial developments, the exploration for effective antimicrobial activity based on PK and pharmacodynamic (PD) properties is the solution [4]. Moreover, the standard recommended dose of antibiotic, which was derived from (1) PK data from healthy volunteers or non-intensive care unit (ICU) patients, and (2) the minimum inhibitory concentration (MIC) for non-resistant bacteria, may increase the risk of treatment failure [4]. Roberts et al. performed a PK study of 8 beta-lactam antibiotics in critically ill patients across 68 ICUs in 10 European countries. From a total of 248 patients, 16% of patients did not achieve 50% of the time of free antibiotic concentrations above the minimum inhibitory concentration (fT>MIC) of the pathogen, and these patients were less likely to complete a treatment course and achieve positive clinical outcome with the initially selected beta-lactam [8].

Meropenem, a member of the carbapenem class, is widely used as empirical therapy in the treatment of sepsis and septic shock regarding its broad-spectrum activity and a low toxicity profile. The PD of meropenem is a time-dependent activity, similar to other beta-lactam antibiotics. At least 40% fT>MIC is required for carbapenems to achieve their bactericidal activity [4]. However, to achieve favorable microbiological and clinical outcomes for critically ill patients with severe infections, achievement of 100% fT>MIC is required, and prolonged infusion of meropenem is recommended [6, 9]. Additionally, the standard recommended dose of meropenem was not sufficient to achieve a higher PK/PD target in sepsis and septic shock patients [4, 10, 11]. Jaruratanasirikul et al. conducted a population PK study of meropenem in critically ill patients. They also indicated that the maximum recommended dose of 2 g of meropenem every 8 h may be needed for an early phase of sepsis and septic shock [12].

Currently, there was a lack of study of the high-dose meropenem in sepsis and septic shock patients. We proposed that the given high-dose, prolonged infusion regimen in early phase sepsis and septic shock would help reduce mortality, defined by a reduction in modified sequential organ failure assessment (mSOFA) score. Therefore, we designed a randomized study to compare the changes in mSOFA score and other clinical outcomes of the standard-dose versus the high-dose meropenem in critically ill patients with sepsis and septic shock.

## Methods

### Design and settings

This study was a prospective, single-center, randomized, open-label study conducted at an academic hospital in Bangkok, Thailand. This trial was registered in ClinicalTrial.gov identifier NCT03344627 and was approved by the committee on human rights related to research, Faculty of Medicine Ramathibodi Hospital, Mahidol University.

### Inclusion and exclusion criteria

The inclusion criteria included (1) patients aged > 18 years, (2) patients diagnosed as sepsis and septic shock according to sepsis-3 criteria and received meropenem within 1 h after diagnosis, (3) patients (or relatives) signing the consent form. The exclusion criteria included (1) patients having sepsis with central nervous system infection, infective endocarditis; (2) patients requiring surgical condition within 72 h after randomization; (3) patients receiving extracorporeal membrane oxygenation (ECMO) within 3 days after randomization; (4) patients with active seizure; (5) patients receiving meropenem within 1 week prior to randomization; (6) pregnancy or lactation; (7) patients having known allergy to meropenem; and (8) patients receiving meropenem as empirical therapy less than 3 days.

### Patient randomization and treatment protocol

Patients were randomized using a sealed opaque envelope in a block of four stratified according to their status prior to ICU admission (escalated from other wards or admitted to ICU from emergency department (ED)). They were assigned to receive either standard-dose meropenem (the standard-dose group, 1 g of meropenem intravenous (IV) infused over 30 min, then 1 g of meropenem IV infused over 3 h every 8 h) or high-dose meropenem (the high-dose group, 2 g of meropenem IV infused over 30 min then 2 g of meropenem IV infused over 3 h every 8 h). The study was an open-label study in which the dose was assigned according to the randomization. The enrolled patients were treated using a standard supportive treatment in accordance with the assignment of the meropenem group. The investigator had not been involved in other treatments. Creatinine clearance (ClCr) was calculated by the Cockcroft formula. The dosage of meropenem during the study period was adjusted according to ClCr. It should be noted that patients were allowed to receive concomitant antimicrobial for polymicrobial infections. De-escalation should be narrower spectrum antibiotics according to patient-specific microbiological culture. The recommended duration of antibiotic treatment was based on the decision of the ICU physician team.

### Data collection and clinical endpoints

Baseline characteristics such as age, gender, weight, type of admission, Acute Physiology and Chronic Health Evaluation II (APACHE II), mSOFA scores, concomitant antimicrobial therapy, and source of infection were collected at the start of meropenem therapy.

The primary outcome was delta mSOFA scores (differences between mSOFA scores at day 4 after randomization and mSOFA score at day 1 after randomization). Secondary outcomes were 14-day mortality, 28-day mortality, clinical cure, microbiological cure, vasopressor-free days, ventilator-free days, ICU- and hospital-free days, and safety of both dosing regimen groups. Clinical cure was defined as a complete or partial resolution of fever (temperature > 38.3 °C) for more than 24 h and leucocytosis (white blood cell count > 12 × 10^9^/L). Microbiological cure was defined as eradication of the study entry pathogen at the suspected site(s) of infection within 14 days (collected at day 3, 5, 7, 10, and 14) after giving meropenem. Blood samples for meropenem level were obtained and measured by liquid chromatography-tandem mass spectrometry (LC-MS/MS) to calculate an achievement of the PD target. Superinfection was defined as recently developed infections by the Centers for Disease Control and Prevention (CDC) criteria within 14 days after being given meropenem, or any newly discovered carbapenem-resistant or colistin-resistant gram-negative bacteria. Vasopressor, ventilator, ICU, and hospital-free days were counted as 28 − *X* if successfully liberated from vasopressor use, mechanical ventilation, ICU stay or hospital stay *X* days after the first day of being given meropenem. Safety outcome was recorded daily during the course of antibiotic treatment.

### Statistical analysis

The sample size was calculated based on our preliminary study, the patient who received meropenem and survived from sepsis and septic shock had shown a reduction in delta mSOFA score of 1.850 (standard deviation (SD) of 2.961). To show a reduction in mSOFA score of 2 in the high-dose group, a sample size of 39 patients in each group was deemed necessary with type I error of 5%, power of 80%, and predicted 10% dropout from the study.

The per-protocol approach was adopted to make inference on the primary outcome. The Kolmogorov-Smirnov test was selected for the check of the data distribution. Continuous normally distributed data and non-normally distributed data were tested using unpaired *t* test and Mann-Whitney *U* test, respectively. Categorical data were tested by Chi-square or Fisher’s exact test, as appropriate. Normally distributed data and non-normal distributed data are presented as mean + SD and median with interquartile range. A two-sided *P* value < 0.05 was considered statistically significant for all tests. All statistical calculations were performed with the SPSS statistical package version 18.0.

## Results

**Fig. 1:**
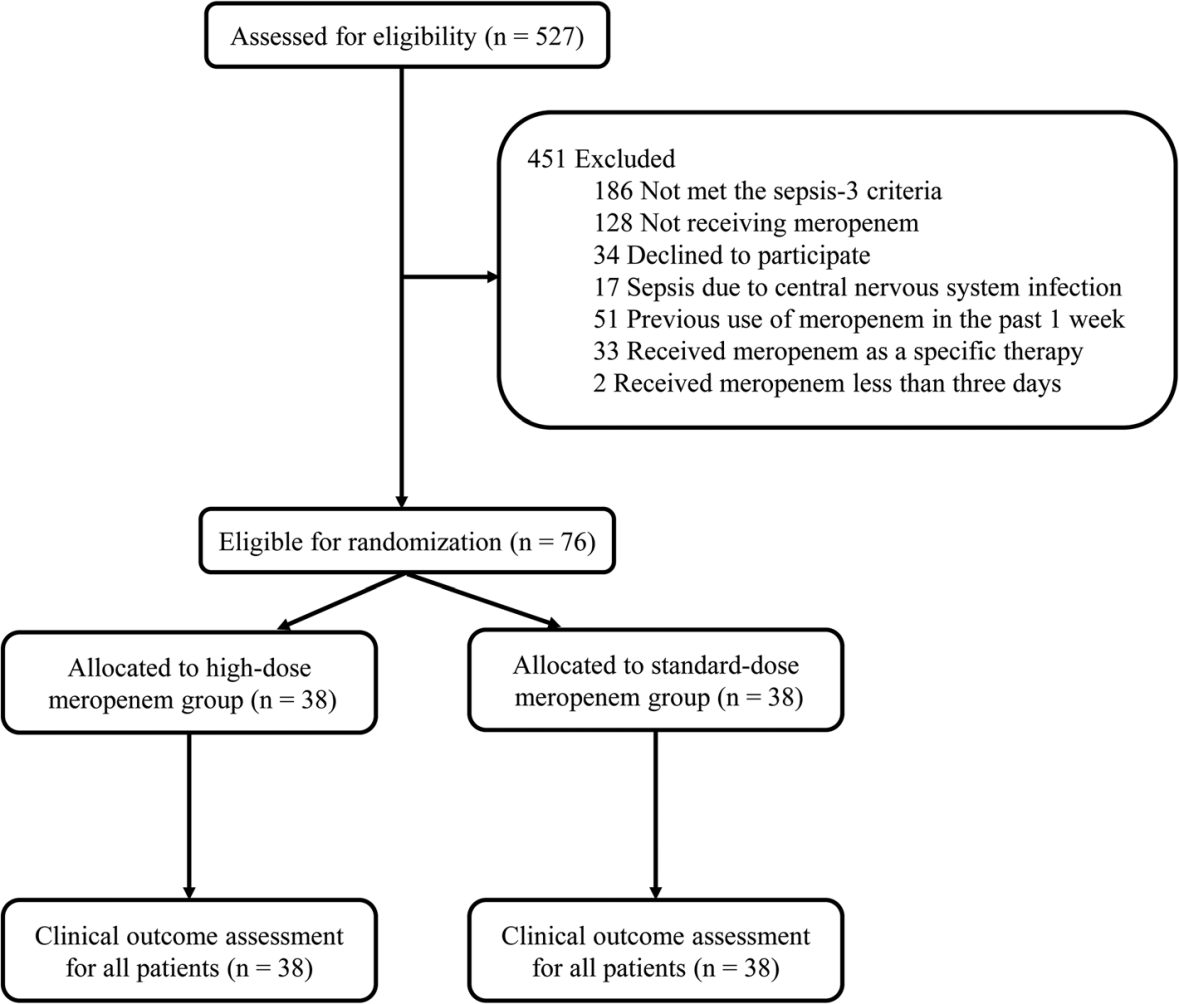
Flow of patients through the trial

Five hundred and twenty-seven patients were screened between August 2017 and October 2018, of whom 451 patients failed to meet the inclusion criteria. The remaining 76 patients were enrolled in the study (Fig. 1). Thirty-eight patients were allocated to each of the standard-dose group and the high-dose group. Age, gender, weight, underlying diseases, ClCr, type of admission, the severity of illness, and source of infection were comparable between the two groups. The major source of infection in the standard-dose meropenem group was respiratory (39.5%), bloodstream (31.6%), multiple sources (13.2%), and urinary tract (5.3%). The main source of infection in the high-dose meropenem group was respiratory (42.1%), followed by urinary tract (23.7%), multiple sources (21.2%), and bloodstream (18.4%) as shown in Table 1.

**Table 1:** Demographics and baseline characteristics

Characteristics	Standard-dose meropenem (*n* = 38)	High-dose meropenem (*n* = 38)	*P* value
Age (years), mean (SD)	67 (14.6)	69 (19.0)	0.657
Male, *n* (%)	21 (55.3)	19 (50)	0.819
Weight (kg), mean (SD)	56.4 (12.4)	57.2 (14.7)	0.797
Underlying disease, *n* (%)			
Diabetes	13 (34.2)	7 (18.4)	0.192
Hypertension	17 (44.7)	15 (39.5)	0.817
Heart failure	5 (13.2)	3 (7.9)	0.711
Chronic kidney disease	5 (13.2)	5 (13.2)	1.000
End stage renal disease	6 (15.8)	1 (2.6)	0.108
Chronic liver disease	4 (10.5)	3 (7.9)	1.000
COPD	8 (21.1)	2 (5.3)	0.086
Hematologic malignancy	7 (18.4)	8 (21.1)	1.000
Solid malignancy	8 (21.1)	7 (18.4)	1.000
Neutropenia	4 (10.5)	7 (18.4)	0.516
ARDS	13 (34.2)	10 (26.3)	0.628
Cockroft-Gout ClCr (mL/min), median (IQR)	41.81 (7.36–166.8)	30.37 (7.04–141.75)	0.377
Type of admission, *n* (%)			
ED	17 (44.7)	18 (47.4)	1.000
Ward	21 (55.3)	20 (52.6)	
Mechanical ventilator, *n* (%)	29 (76.3)	27 (71.1)	0.795
APACHE II score, mean (SD)	18.8 (5.7)	20 (6.3)	0.395
mSOFA score, median (IQR)	7 (0-13)	8 (1-16)	0.088
Septic shock, *n* (%)	26 (68.4)	27 (71.1)	1.000
Source of infection			
Unknown, *n* (%)	9 (23.7)	6 (15.8)	0.565
Respiratory, *n* (%)	15 (39.5)	16 (42.1)	1.000
Urinary tract, *n* (%)	2 (5.3)	9 (23.7)	0.047
Bloodstream, *n* (%)	12 (31.6)	7 (18.4)	0.289
Multiple sources, *n* (%)	5 (13.2)	8 (21.2)	0.540
Culture, *n* (%)	21 (55.3)	23 (60.5)	0.817
Polymicrobial	6 (28.6)	3 (13.0%)	0.272
Gram-positive pathogens	5 (23.8)	1 (4.3)	0.088
Gram-negative pathogens	16 (76.2)	20 (87.0)	0.448
*Escherichia coli*	7 (33.3)	6 (26.1)	0.744
*Klebsiella pneumoniae*	8 (38.1)	7 (30.4)	0.752
*Pseudomonas aeruginosa*	1 (4.8)	6 (26.1)	0.097
*Acinetobacter baumannii*	1 (4.8)	1 (4.3)	1.000

*COPD* chronic obstructive pulmonary disease, *ARDS* acute respiratory distress syndrome, *ED* emergency department, *APACHE II* Acute Physiology and Chronic Health Evaluation II, *mSOFA* modified Sequential Organ Failure Assessment

The median duration of meropenem treatment was significantly higher in the standard-dose group (6 (3 to 16) days vs. 5 (3 to 21) days; *P* value = 0.035). Concomitant antimicrobial therapy was used in 42.1% in the standard-dose group and 50% in the high-dose group. The antibacterial drugs comprised of multiple classes, such as polymyxin, glycopeptides, aminoglycosides, and fluoroquinolones. The types of concomitant antibiotics were not significantly different between groups (Table 2). De-escalations from meropenem to narrow-spectrum antibiotics were more than 50% in both groups.

**Table 2:** Duration of meropenem and concomitant antimicrobial therapy

	Standard-dose meropenem (*n* = 38)	High-dose meropenem (*n* = 38)	*P* value
Day of meropenem therapy, median (IQR)	6 (3–16)	5 (3–21)	0.035*
Concomitant antimicrobial therapy, *n* (%)	16 (42.1)	19 (50)	0.646
Colistin, *n* (%)	3 (18.8)	2 (10.5)	0.642
Vancomycin, *n* (%)	7 (43.8)	6 (31.6)	0.503
Cotrimoxazole, *n* (%)	1 (6.3)	4 (21.1)	0.347
Levofloxacin, *n* (%)	0 (0)	3 (15.8)	0.234
Amikacin, *n* (%)	4 (25)	3 (15.8)	0.677
Azithromycin, *n* (%)	1 (6.3)	2 (10.5)	1.000
Oseltamivir, *n* (%)	2 (12.5)	2 (10.5)	1.000
De-escalation, *n* (%)	21 (55.3)	25 (65.8)	0.436

*Statistically significant difference

**Fig. 2:**
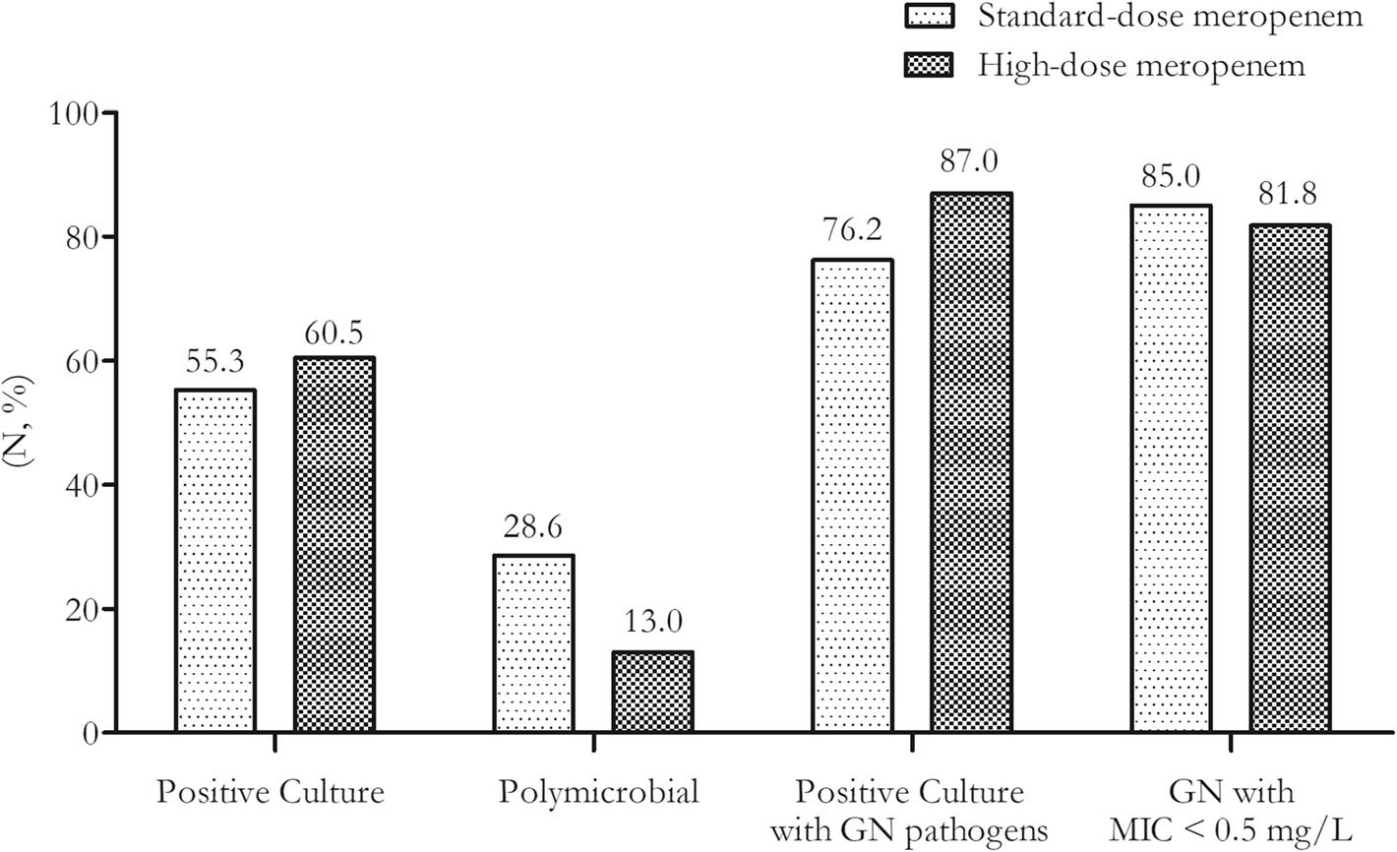
Bacterial isolates. GN, gram-negative bacteria; MIC, minimum inhibitory concentration

The microbiological culture was positive in 55.3% in the standard-dose group and 60.5% in the high-dose group. There was no significant difference regarding culture positive for polymicrobial, gram-positive bacteria and gram-negative bacteria (Table 1). Similar results for gram-negative bacterial isolates showed no difference, and more than 80% were susceptible to meropenem (Fig. 2). The most common organisms were *Enterobacteriaceae*, *Pseudomonas aeruginosa,* and *Acinetobacter baumannii*, respectively, in patients with culture-positive gram-negative bacteria (Table 1).

Delta mSOFA score was comparable between the high-dose and the standard-dose group (− 1 (− 9 to 4) vs. − 1 (− 6 to 3); *P* value = 0.746). Delta cardiovascular score was decreased (1-point reduction) in the high-dose group compared to no change (no point reduction) in the standard-dose group, but this was not statistically significant. For the secondary outcome, the clinical cure rate was not different (86.8% in the high-dose vs. 86.8% in the standard-dose group; *P* value = 1.00). Lower 14-day mortality and 28-day mortality were observed in the high-dose group (10.5% vs. 15.8%; *P* value = 0.736) and (34.2% vs. 44.7%; *P* value = 0.482), respectively. The microbiological cure had a trend to better cure rate in the high-dose group than the standard-dose group (55.3% vs. 44.7%; *P* value = 0.492). Vasopressor-free days, ventilator-free days, ICU-free days, and hospital-free days were not significantly different between the treatment groups (Table 3). Meropenem levels were obtained from eighteen patients (8 patients in the standard-dose group and 10 patients in the high-dose groups). Regarding the identified MICs, PD target of 40% of the time above identified MIC was entirely achieved in all patients. However, the PD target of 100% of the time above the identified MIC was achieved in 66.7% (2/8 patients) in the standard-dose group and 100% (5/5 patients) in the high-dose group with no statistically significant difference between groups (*P* value *=* 0.375) (Table 4).

**Table 3:** Primary and secondary outcomes

Outcomes	Standard-dose meropenem (*n* = 38)	High-dose meropenem (*n* = 38)	*P* value
**Primary outcome**			
Delta mSOFA score, median (IQR)	− 1 (− 6 to 3)	− 1 (− 9 to 4)	0.746
Delta respiratory score	− 1 (− 3 to 2)	− 1 (− 4 to 2)	0.136
Delta cardiovascular score	0 (− 4 to 3)	− 1 (− 4 to 3)	0.599
Delta liver score	0 (− 1 to 2)	0 (− 1 to 2)	0.570
Delta renal score	0 (− 1 to 2)	0 (− 2 to 1)	0.201
Delta coagulation score	0 (− 2 to 3)	0 (− 1 to 3)	0.052
**Secondary outcomes**			
14-day mortality, *n* (%)	6 (15.8)	4 (10.5)	0.736
28-day mortality, *n* (%)	17 (44.7)	13 (34.2)	0.482
Clinical cure, *n* (%)	33 (86.8)	33 (86.8)	1.000
Microbiological cure, *n* (%)	17 (44.7)	21 (55.3)	0.492
Vasopressor-free days, median (IQR)	24.5 (0–27)	25 (0–27)	0.350
Ventilator-free days, median (IQR)	11.5 (0–24)	10.0 (0–28)	0.819
ICU-free days, median (IQR)	0.5 (0–19)	9.5 (0–20)	0.819
Hospital-free days, median (IQR)	0 (0–74)	37.5 (0–72)	0.819
Adverse events			
Diarrhea, *n* (%)	12 (31.6)	8 (21.1)	0.435

*mSOFA* modified sequential organ failure assessment score, *ICU* intensive care unit

**Table 4:** Pharmacodynamic target of the time above the minimum inhibitory concentration

MIC (mg/L)	Standard-dose meropenem (*n* = 8)	High-dose meropenem (*n* = 10)
Identified MIC, *n*	3	5
• Achievement of 40%T>MIC, *n* (%)	3 (100%)	5 (100%)
• Achievement of 100%T>MIC, *n* (%)	2 (66.7%)	5 (100%)
MIC = 8, *n*	8	10
• Achievement of 40%T>MIC, *n* (%)	5 (62.5%)	9 (90%)
• Achievement of 100%T>MIC, *n* (%)	2 (25%)	4 (40%)

*MIC* minimum inhibitory concentration, *%T>MIC* percent of the time of antibiotic concentrations above the minimum inhibitory concentration

**Fig. 3:**
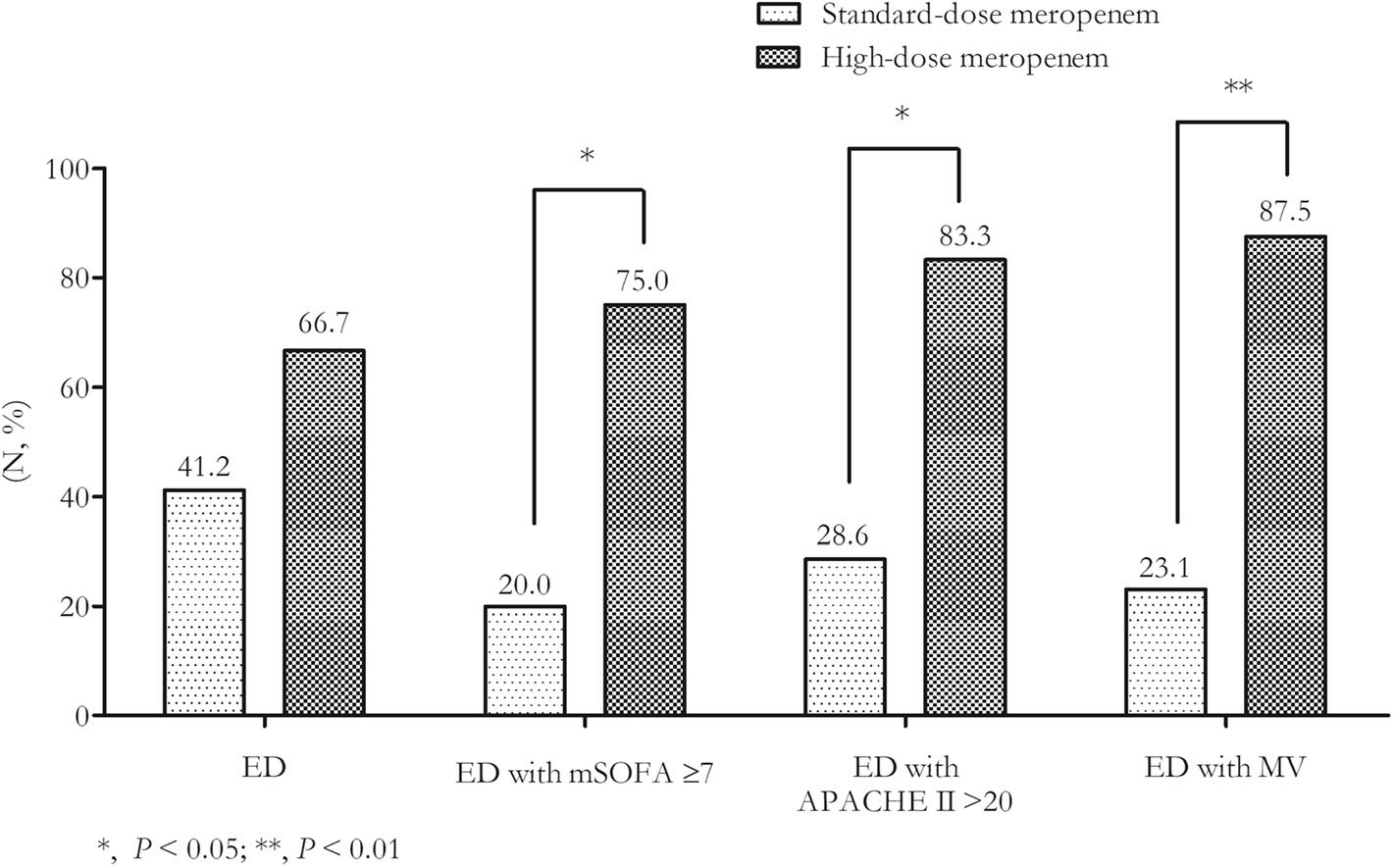
Comparison of microbiological cure rate in patients who admitted from the emergency department. ED, emergency department; mSOFA, modified sequential organ failure assessment score; APACHE II, Acute Physiology and Chronic Health Evaluation II; MV, mechanical ventilation

In subgroup analysis, there were no clinical and microbiological differences in outcomes in sepsis and septic shock patients who escalated from other wards to ICU. However, patients who admitted from ED had a higher trend of microbiological cure rate in the high-dose group (66.7% vs. 41.2%; *P* value = 0.181). In patients admitted from ED with mSOFA score > 7, high-dose meropenem demonstrated significantly better microbiological cure compared with standard-dose (75% (9/12) vs. 20% (2/10); *P* value = 0.03). Likewise, in patients admitted from ED and had APACHE II score > 20, the high-dose group had a higher microbiological cure rate (83.3% (10/12) vs. 28.6% (2/7); *P* value = 0.045) than the standard-dose group. In patients admitted from ED who used the mechanical ventilator, the high-dose group had a higher microbiological cure rate than the standard-dose group (87.5% (7/8) vs. 23.1% (3/13); *P* value = 0.008) (Fig. 3). Moreover, even though urinary tract infections (UTI) were higher in the high-dose group (23.7%) than in the standard-dose group (5.3%), *P* value = 0.047, they did not affect any significant changes in both primary and secondary outcomes in bivariate analysis.

For adverse events, diarrhea was reported in 12 patients (31.6%) in the standard-dose group and 8 patients (21.1%) in the high-dose group with no statistical significance (*P* value = 0.435). However, there were no other adverse events, such as seizure or *Clostridium difficile* infection, found in the two groups, as shown in Table 3.

## Discussion

In our study, we found no difference in delta mSOFA between the standard-dose and the high-dose meropenem group. In addition, each component of the SOFA score was not worsening in both of the standard-dose and the high-dose groups. The explanation of this comparable delta mSOFA between the standard-dose and the high-dose meropenem might be as follows. First, the pathophysiology of sepsis and septic shock starts from dysregulated host responses to infection. A combination of hemodynamic and cellular failures were then followed constantly [13]. Appropriate antibiotic therapy at an early point of sepsis diagnosis will help in reducing microbial load. Consequently, the burden from microbial toxin released or produced by bacteria will be terminated [14]. Inflammatory response leading to tissue injury would then be declined [14]. Since critically ill patients have substantially physiologic changes, the suboptimal concentration of antibiotics was reported [8, 15, 16]. Thus, high-dose antibiotics should be taken into consideration to achieve better clinical outcomes [6]. Nevertheless, cellular failure due to the individual dysregulated host responses to pro-inflammatory cytokine and alteration of cellular immune function may also progress and might not have been helped by the higher dose of antibacterial [13, 17]. Second, 44.7% of patients included in our study had a negative culture, which might have been from non-infectious causes, viruses, or fungal infection [18, 19]. In fact, supportive treatments such as fluid resuscitation, vasopressor therapy, and nursing care were appropriate in both of the standard-dose and the high-dose groups.

For the secondary outcomes, 14-day and 28-day mortality, clinical cure, and vasopressor-, ventilator-, ICU-, and hospital-free days, there were no significant differences between the two meropenem dosing strategies. Since the sample size in our study was explicitly calculated focused on delta mSOFA score, the sample size might be insufficiently powered to detect differences of secondary outcomes between two groups. Besides, even though there was a trend of higher ICU- and hospital-free days in the high-dose group, there was no statistical difference between the groups. The secondary outcomes’ results might have been confounded by a persistent inflammation-immunosuppression and catabolism syndrome (PICS), and post-sepsis syndrome which are increasingly found in critically ill patients [20, 21]. Therefore, increased risk of death and reduced health-related quality of life would be associated with long-term effect consequences reflecting the secondary outcomes [21]. Moreover, serious adverse drug events, seizure, and *Clostridium difficile* infection were not reported in the high-dose group. Since we excluded patients who might possibly have risk for potential toxicity from meropenem accumulation and we adjusted the meropenem dosing regimen by patients’ renal functions, no dose-related serious adverse events was found.

Although the duration of prescribed meropenem in the standard-dose group was significantly shorter than the high-dose group (5 days vs. 6 days; *P* value = 0.035), the high-dose group had still shown a trend toward increasing in microbiological cure compared to the standard-dose group (55.3% vs. 44.7%; *P* value = 0.492). This was because the higher concentration of meropenem at the site of infection was required in order to get a better microbiological cure [22, 23]. Regarding the meropenem concentration in our study, both regimens of meropenem achieved PD target of 40% of the time above the identified MIC. Considering suspected resistant bacteria, achieving the PD target of 40%T>MIC of 8 mg/L was higher in the high-dose group (9/10 patients, 90%) compared with the standard-dose group (5/8 patients, 62.5%). Likewise, achieving the PD target of 100%T>MIC was higher in the high-dose group (4/10 patients, 40%) compared to the standard-dose group (2/8 patients, 25%). Since there was a small number of blood samples collected, statistical differences were not found. Frippiat et al. performed Monte Carlo simulation to estimate meropenem concentration at epithelial lining fluid (ELF) for meropenem 2 g every 8 h and 1 g every 8 h. At MIC 0.5 mg/L, which was the most cut point MIC in our study, prolonged infusions of meropenem 2 g every 8 h demonstrated ≥ 90% probability of target attainment (PTA) of 100%T>MIC. While meropenem 1 g every 8 h demonstrated ≥ 90% PTA of 40%T>MIC [24]. Consequently, an increasing meropenem plasma concentration in the high-dose group might assumingly have an increasing trend in meropenem concentration in ELF, which was a target site in almost half of the patients in our study. Nevertheless, we still did not find statistically significant difference between the two groups regarding microbiological cure.

Therefore, we performed subgroup analysis, which showed there were 3 groups of patients that would benefit from the high-dose meropenem. In patients admitted from the ED with mSOFA score > 7, the high-dose group demonstrated significantly better microbiological cure compared with the standard-dose group (75% (9/12 patients) vs. 20% (2/10 patients); *P* value = 0.03). Similarly, in patients admitted from the ED with an APACHE II score > 20 and patients admitted from the ED with mechanical ventilators, the high-dose group had a higher microbiological cure rate than the standard-dose group (83.3% (10/12 patients) vs. 28.6% (2/7 patients); *P* value = 0.045 and 87.5% (7/8 patients) vs. 23.10% (3/13 patients); *P* value = 0.008). Since this is the first study identifying different outcomes between two meropenem dosing regimens in ED, high-severity patients might get better outcomes from the higher dose of meropenem. The reason for better microbiological cure might be from higher tissue penetration and overall maintenance of plasma concentration in severe patients. Goncalves et al. found that critically ill patients with a greater baseline severity had a significant increase in the volume of distribution, leading to low meropenem concentrations. Decreased antibiotic concentration at the infection site was eventually found from reduced microvascular perfusion in critically ill patients [25, 26]. However, the high-dose meropenem did not demonstrate better clinical outcomes, delta mSOFA, mortality, vasopressor-free days, ventilator-free days, ICU-free days, and hospital-free days, in these 3 groups of patients.

However, our study has several limitations. Firstly, since this was the first study comparing 3 g/day with 6 g/day of meropenem, the sample size calculation was based on the delta mSOFA from the previous 3 months before patient recruitment, but not from the differences of delta mSOFA between two meropenem dosing. Therefore, the sample size might not have been enough to detect significances between the two dosing strategies. Second, the negative culture found in about 50% of patients in our study, impact on different meropenem dosing could not be assumed. Early differentiation of non-bacterial infection from bacterial infection before empirical therapy has not yet been easily done in our institution. Third, almost all of our patients’ relatives experienced grief when confronted with a deteriorating phase of their love ones. Informed consent regarding blood sample collection was too difficult when there was a treatment limitation decision. For that reason, meropenem blood concentration collection was performed in only 5% of our recruited patients. Lastly, pathogens identified in this study had relatively low MICs. Narrowing the group of patients that might benefit from the high-dose group might be an option. Patient medical history, local antibiogram, and epidemiology of antimicrobial resistance in each area might be needed to modify inclusion criteria in order to specify the group of patients likely to be infected by higher MICs.

## Conclusions

Empirical therapy with a high-dose of meropenem in critically ill patients showed no statistical difference in patient’s clinical outcomes compared to a standard-dose of meropenem. However, subgroup analysis showed that patients who were admitted from ED and had mSOFA score ≥ 7 or APACHE II score > 20 or had been on mechanical ventilator manifested superior microbiological cure rate in sepsis or septic shock patients admitted from ED.

## Data Availability

The datasets used and/or analyzed during the current study are available from the corresponding author on reasonable request.

## Abbreviations

mSOFA: Modified sequential organ failure assessment score
ED: Emergency department
PK: Pharmacokinetic
MDR: Multidrug-resistant
ESBL: Extended-spectrum beta-lactamase
PD: Pharmacodynamic
ICU: Intensive care unit
fT>MIC: Time of free antibiotic concentrations above the minimum inhibitory concentration
ClCr: Creatinine clearance
APACHE II: Acute Physiology and Chronic Health Evaluation II
LC-MS/MS: Liquid chromatography-tanden mass spectrometry
CDC: Centers for Disease Control and Prevention
SD: Standard deviation
PTA: Probability of target attainment
ELF: Epithelial lining fluid

## References

[1] Cecconi M, Evans L, Levy M, Rhodes A (2018). Sepsis and septic shock. Lancet..

[2] Ulldemolins M, Vaquer S, Llauradó-Serra M, Pontes C, Calvo G, Soy D (2014). Beta-lactam dosing in critically ill patients with septic shock and continuous renal replacement therapy. Crit Care..

[3] Roberts JA, Kumar A, Lipman J (2017). Right dose, right now: customized drug dosing in the critically ill. Critical care medicine..

[4] Delattre IK, Taccone FS, Jacobs F, Hites M, Dugernier T, Spapen H (2017). Optimizing β-lactams treatment in critically-ill patients using pharmacokinetics/pharmacodynamics targets: are first conventional doses effective?. Expert review of anti-infective therapy..

[5] Blot SI, Pea F, Lipman J (2014). The effect of pathophysiology on pharmacokinetics in the critically ill patient—concepts appraised by the example of antimicrobial agents. Advanced drug delivery reviews..

[6] Masich AM, Heavner MS, Gonzales JP, Claeys KC (2018). Pharmacokinetic/pharmacodynamic considerations of beta-lactam antibiotics in adult critically ill patients. Current infectious disease reports..

[7] Suwantarat N, Carroll KC (2016). Epidemiology and molecular characterization of multidrug-resistant Gram-negative bacteria in Southeast Asia. Antimicrobial Resistance Infection Control..

[8] Roberts JA, Paul SK, Akova M, Bassetti M, De Waele JJ, Dimopoulos G (2014). DALI: defining antibiotic levels in intensive care unit patients: are current β-lactam antibiotic doses sufficient for critically ill patients?. Clinical infectious diseases..

[9] Rizk NA, Kanafani ZA, Tabaja HZ, Kanj SS (2017). Extended infusion of beta-lactam antibiotics: optimizing therapy in critically-ill patients in the era of antimicrobial resistance. Expert review of anti-infective therapy..

[10] Burger R, Guidi M, Calpini V, Lamoth F, Decosterd L, Robatel C (2018). Effect of renal clearance and continuous renal replacement therapy on appropriateness of recommended meropenem dosing regimens in critically ill patients with susceptible life-threatening infections. Journal of Antimicrobial Chemotherapy..

[11] Sjövall F, Alobaid AS, Wallis SC, Perner A, Lipman J, Roberts JA (2017). Maximally effective dosing regimens of meropenem in patients with septic shock. J Antimicrobial Chemother.

[12] Jaruratanasirikul S, Thengyai S, Wongpoowarak W, Wattanavijitkul T, Tangkitwanitjaroen K, Sukarnjanaset W (2015). Population pharmacokinetics and Monte Carlo dosing simulations of meropenem during the early phase of severe sepsis and septic shock in critically ill patients in intensive care units. Antimicrobial agents and chemotherapy..

[13] Lelubre C, Vincent J-L (2018). Mechanisms and treatment of organ failure in sepsis. Nature Reviews Nephrology..

[14] Kumar A (2014). An alternate pathophysiologic paradigm of sepsis and septic shock: implications for optimizing antimicrobial therapy. Virulence..

[15] Ehmann L, Zoller M, Minichmayr IK, Scharf C, Maier B, Schmitt MV (2017). Role of renal function in risk assessment of target non-attainment after standard dosing of meropenem in critically ill patients: a prospective observational study. Crit Care..

[16] Wu C-C, Tai C-H, Liao W-Y, Wang C-C, Kuo C-H, Lin S-W (2019). Augmented renal clearance is associated with inadequate antibiotic pharmacokinetic/pharmacodynamic target in Asian ICU population: a prospective observational study. Infection Drug Resistance..

[17] Delano MJ, Ward PA (2016). The immune system's role in sepsis progression, resolution, and long-term outcome. Immunological reviews..

[18] de Prost N, Razazi K, Brun-Buisson C (2013). Unrevealing culture-negative severe sepsis. Crit Care..

[19] Vincent J-L, Sakr Y, Sprung CL, Ranieri VM, Reinhart K, Gerlach H (2006). Sepsis in European intensive care units: results of the SOAP study. Critical care medicine..

[20] Mira JC, Gentile LF, Mathias BJ, Efron PA, Brakenridge SC, Mohr AM (2017). Sepsis pathophysiology, chronic critical illness and PICS. Crit Care Med.

[21] Mostel Z, Perl A, Marck M, Mehdi SF, Lowell B, Bathija S (2020). Post-sepsis syndrome–an evolving entity that afflicts survivors of sepsis. Mol Med.

[22] Yu Z, Pang X, Wu X, Shan C, Jiang S (2018). Clinical outcomes of prolonged infusion (extended infusion or continuous infusion) versus intermittent bolus of meropenem in severe infection: A meta-analysis. PloS one..

[23] Liang SY, Kumar A (2015). Empiric antimicrobial therapy in severe sepsis and septic shock: optimizing pathogen clearance. Curr Infectious Dis Rep.

[24] Frippiat F, Musuamba FT, Seidel L, Albert A, Denooz R, Charlier C (2015). Modelled target attainment after meropenem infusion in patients with severe nosocomial pneumonia: the PROMESSE study. The Journal of antimicrobial chemotherapy..

[25] Goncalves-Pereira J, Silva NE, Mateus A, Pinho C, Povoa P (2014). Assessment of pharmacokinetic changes of meropenem during therapy in septic critically ill patients. BMC Pharmacol Toxicol.

[26] Gonçalves-Pereira J, Póvoa P (2011). Antibiotics in critically ill patients: a systematic review of the pharmacokinetics of β-lactams. Crit Care.

